# Supplementary material for: Iterative improvement in the automatic modular design of robot swarms
Source: PeerJ Comput Sci. 2020 Dec 7;6:e322. doi: 10.7717/peerj-cs.322 (PMC7924708; doi:10.7717/peerj-cs.322)
Supplement: Supplemental Information 3 [file peerj-cs-06-322-s003.zip › argos3/doc/api/standalone/a00576_source.html]

ARGoS: plugins/simulator/entities/light\_entity.cpp Source File


- Main Page
- Related Pages
- Namespaces
- Classes
- Files

- File List
- File Members

# plugins/simulator/entities/light\_entity.cpp

Go to the documentation of this file.

```
00001 
00007 #include "light_entity.h"
00008 #include <argos3/core/simulator/simulator.h>
00009 #include <argos3/core/simulator/space/space.h>
00010 #include <argos3/plugins/simulator/media/led_medium.h>
00011 
00012 namespace argos {
00013 
00014    /****************************************/
00015    /****************************************/
00016 
00017    CLightEntity::CLightEntity() :
00018       CLEDEntity(NULL),
00019       m_fIntensity(1.0f) {}
00020       
00021    /****************************************/
00022    /****************************************/
00023 
00024    CLightEntity::CLightEntity(const std::string& str_id,
00025                               const CVector3& c_position,
00026                               const CColor& c_color,
00027                               Real f_intensity) :
00028       CLEDEntity(NULL,
00029                  str_id,
00030                  c_position,
00031                  c_color),
00032       m_fIntensity(f_intensity) {}
00033 
00034    /****************************************/
00035    /****************************************/
00036 
00037    void CLightEntity::Init(TConfigurationNode& t_tree) {
00038       try {
00039          /* Init parent */
00040          CLEDEntity::Init(t_tree);
00041          /* Parse XML */
00042          GetNodeAttribute(t_tree, "intensity", m_fIntensity);
00043          std::string strMedium;
00044          GetNodeAttribute(t_tree, "medium", strMedium);
00045          CLEDMedium& cLEDMedium = CSimulator::GetInstance().GetMedium<CLEDMedium>(strMedium);
00046          cLEDMedium.AddEntity(*this);
00047       }
00048       catch(CARGoSException& ex) {
00049          THROW_ARGOSEXCEPTION_NESTED("Error while initializing light entity", ex);
00050       }
00051    }
00052 
00053    /****************************************/
00054    /****************************************/
00055 
00056    REGISTER_ENTITY(CLightEntity,
00057                    "light",
00058                    "Carlo Pinciroli [ilpincy@gmail.com]",
00059                    "1.0",
00060                    "A colored light.",
00061                    "The light entity is an entity that emits a light detectable by a robot camera\n"
00062                    "(as a normal LED) or by light sensors. A light is bodyless, therefore it must\n"
00063                    "not be added to physics engines.\n"
00064                    "A light is characterized by a color and an intensity. The color of the light\n"
00065                    "is perceived by cameras. The intensity modifies the reading of the light\n"
00066                    "sensors. The higher the intensity, the closer the light is perceived.\n\n"
00067                    "REQUIRED XML CONFIGURATION\n\n"
00068                    "  <arena ...>\n"
00069                    "    ...\n"
00070                    "    <light id=\"light0\"\n"
00071                    "           position=\"0.4,2.3,0.25\"\n"
00072                    "           orientation=\"0,0,0\"\n"
00073                    "           color=\"yellow\"\n"
00074                    "           intensity=\"1.0\"\n"
00075                    "           medium=\"leds\"/>\n"
00076                    "    ...\n"
00077                    "  </arena>\n\n"
00078                    "The 'id' attribute is necessary and must be unique among the entities. If two\n"
00079                    "entities share the same id, initialization aborts.\n"
00080                    "The 'position' attribute specifies the position of the center of the light.\n"
00081                    "The attribute values are in the X,Y,Z order.\n"
00082                    "The 'orientation' attribute specifies the orientation of the light. At the\n"
00083                    "moment this attribute is mandatory but its value is ignored. In the future,\n"
00084                    "it will be used to implement a directional light.\n"
00085                    "The 'color' attribute specifies the color of the light.\n"
00086                    "The 'intensity' attribute sets the intensity of the light. When the value is\n"
00087                    "1.0, the light emits a normal amount of light. When it is 0.5 the amount of\n"
00088                    "light is half, and when the value is 2.0 the emission is doubled. The\n"
00089                    "intensity of the light affects the readings of the light sensors but not\n"
00090                    "those of the cameras.\n"
00091                    "The 'medium' attribute is used to add the light the corresponding LED medium.\n\n"
00092                    "OPTIONAL XML CONFIGURATION\n\n"
00093                    "None.\n",
00094                    "Usable"
00095       );
00096 
00097    /****************************************/
00098    /****************************************/
00099 
00100    REGISTER_STANDARD_SPACE_OPERATIONS_ON_ENTITY(CLightEntity);
00101 
00102    /****************************************/
00103    /****************************************/
00104 
00105 }
```

---

Generated on 10 Jul 2018 for ARGoS by 
 1.6.1
